# Females’ Engagement in Offline and Online Sexual Offending and Their Interactions With the Criminal Justice System: A Gender and Age Comparison

**DOI:** 10.1177/08862605241299445

**Published:** 2024-11-26

**Authors:** Isabelle Hull, Larissa S. Christensen, Nadine McKillop, Susan Rayment-McHugh

**Affiliations:** 1 University of the Sunshine Coast, Maroochydore, Australia

**Keywords:** female-perpetrated sexual offending, child sexual abuse material, technology-facilitated sexual violence, female sex offenders, child exploitation material, female offenders, sexual violence

## Abstract

This study aimed to extend limited extant knowledge of female-perpetrated sexual offenses, including child sexual abuse material (CSAM) offenses, that enter the criminal justice system. Sexual offenses actioned by the police in one jurisdiction of Australia between 1 January 2012 and 30 June 2021 (*N* = 37,864) were analyzed to explore the prevalence of sexual offenses and types of sexual offenses perpetrated; the relationship between perpetrator gender, age, and offense type; and the relationship between perpetrator gender, age, offense type, and likelihood of law enforcement action (*N* = 34,835). Consistent with previous research, (predominantly adult) males were responsible for most sexual offenses before police. Females were responsible for 12.2% of all offenses over this period, with juvenile females (10–17 years) implicated in a significant proportion (10.2%) of all offenses. In fact, juvenile females were responsible for the majority of assaultive CSAM offenses, whereas juvenile males mostly perpetrated offline child sexual abuse offenses. Regarding adults, there was a minimal, statistically significant difference between gender and offense type. Odds of perpetrating an online assaultive CSAM offense were 20 times higher for juvenile females compared to both adult males and adult females, and 7.69 times higher for juvenile females compared to juvenile males. Finally, for the same offense type, gender and age differentially impacted law enforcement action. For all offense types, enforcement and gravity (e.g., arrest and referral to court) of further action, were significantly lower among all females and juvenile males compared to adult males. Juvenile females were least likely to have any serious action taken. These findings provide a seminal platform from which to expand much-needed research on female-perpetrated offending to inform policy and practice.

Despite sexual violence being a widely researched topic, there is a lack of research on female-perpetrated sexual violence (Ten Bensel et al., 2019). Societal perceptions have hindered the study of female-perpetrated sexual offending (Ten Bensel et al., 2019). Entrenched societal gender roles meant females were perceived as caregivers and nurturers (Darling & Christensen, 2020; Denov, 2004; Saradjian, 2010) and thus, incapable of sexual offending (K. M. Brown & Kloess, 2020). In addition to the study of female-perpetrated sexual violence, we know even less about female-perpetrated child sexual abuse material (CSAM) offending (Bickart et al., 2019). This is alarming given the explosion of the internet and associated technologies, which are providing additional platforms for committing these offenses. Using a very large dataset, we set out to gain a more comprehensive picture of females who perpetrate sexual offenses, including female-perpetrated CSAM offending. Along with bringing recognition to this issue, the resulting knowledge can inform prevention and intervention initiatives and, in turn, reduce female-perpetrated sexual offending and its impacts (Bickart et al., 2019).

Earlier research suggests the study of female-perpetrated sexual offending has also been hindered by it being perceived as less of an occurrence, and issue, compared with male-perpetrated sexual violence (Hetherton, 1999). However, more recent studies reveal that females sexually offend at a much higher rate than was once thought (Cortoni et al., 2016). Cortoni et al.’s (2016) meta-analysis of Western countries found that while 2.2% of offenses reported to law enforcement were perpetrated by females, victimization surveys indicated a prevalence rate of 11.6%. Regardless of the occurrence, female-perpetrated sexual offending can have profound negative impacts on victims (Christensen, 2018a; Cortoni et al. 2016). In fact, research has found victims who had been abused by both males and females declared that abuse perpetrated by females was more psychologically damaging (Denov, 2004). Professionals working with such victims have also found the abuse perpetrated by females can be just as *physically* damaging (Christensen, 2018a). That is, females are capable of inflicting both minor and major injuries when perpetrating sexual offenses (Budd & Bierie, 2020).

The landscape of sexual offending has also changed over time with the emergence of the internet and associated technologies. As a result, the opportunity to engage in various crimes has been drastically simplified (Holt et al., 2020); in particular, the globalization of the internet and associated technologies have provided offenders with the ability to access, possess, distribute, and produce CSAM by providing online platforms that promote anonymity and enable global dissemination (R. Brown & Bricknell, 2018; Henshaw et al., 2015). While there is a paucity of information on the precise magnitude of online CSAM (Krone & Smith, 2017), it is believed that through the internet’s mass communication, social networking, cloud-storage, end-to-end encryption, and file-sharing capabilities, alongside its increased number of subscribers, availability of, and access to, CSAM has proliferated. This, in turn, has increased the number of individuals, both adults and juveniles (10–17 years), being processed by the criminal justice system for CSAM-related offenses (Ibrahim, 2022; Queensland Sentencing Advisory Council, 2017).

Despite the proliferation of CSAM offending, research on CSAM offenses has followed a similar pattern historically, with research largely focusing on the role of male perpetrators. As a result, very little is known about the extent of female-perpetrated CSAM offending (Christensen & Woods, 2024). In addition to female-perpetrated sexual offending, female-perpetrated CSAM offending is likely much more common than once thought. Females can play a major role in the possession, distribution, and production of CSAM (Bickart et al., 2019; Christensen, 2023). In fact, in one earlier study that used an online survey, Seigfried et al. (2008) found that of the 30 individuals who used CSAM (307 respondents in total), the ratio was 2:1 (20 males and 10 females). Research conducted on CSAM offenders in the Philippines, the current global epicenter of online CSAM, found that 87% of cases involve at least one female offender (International Justice Mission [IJM], 2020). Therefore, it is evident that females play a central, although poorly understood role, in CSAM offending (Bickart et al., 2019).

CSAM also has profound effects on victims, which is perpetuated by the ease with which CSAM can be distributed online, and its permanence. Not only are the victims subject to horrific abuse during the production of these materials, but they are also revictimized each time an image or recording is uploaded or viewed (Broadhurst, 2019; Wortley & Smallbone, 2006). It is the continued availability of CSAM images and videos, and their ability to be reuploaded and circulated even after removal, that makes CSAM victimization a unique form of trauma (Canadian Centre for Child Protection, 2017; National Center for Missing & Exploited Children, 2019). Of major concern is that female-perpetrated CSAM is most often the female targeting their own child (Bickart et al., 2019). It is in these instances where professionals have suggested victims can find the sexual abuse even more damaging than male-perpetrated abuse due to the deep sense of betrayal committed by the victim’s mother (Christensen, 2018a).

With the number of juveniles engaging socially on digital media platforms, the problem of “sexting” and the risks of non-consensual sharing of sexual images has become an emerging issue. Juveniles can be criminally prosecuted for engaging in sexting as the law often treats this behavior within the same category as CSAM offending (Moritz & Christensen, 2020). Research has found that compared with adolescent males, adolescent females are at around a 70% higher risk of coercive sexting victimization (Kernsmith et al., 2018). In fact, research indicates over one-third of Australian girls aged 14 to 17 years have sent, been asked to, or have asked, shared, or shown nude or mostly nude videos and images (U.K. Safer Internet Centre et al., 2017). Therefore, with the transformative and changing uses of technology by young people (Moritz & Christensen, 2020), we will likely see a continued increase in juveniles captured under CSAM legislation.

Given these emerging contexts, and using police data, the present study aimed to further current knowledge of female-perpetrated sexual offenses that enter the criminal justice system. In particular, we sought to learn more about the prevalence of sexual offending and types of sexual offenses perpetrated; whether there is a relationship between perpetrator gender, age, and types of offenses perpetrated; and whether there is a relationship between perpetrator gender, age, types of offenses, and likelihood of action taken by law enforcement. In doing so, we can gain a more comprehensive picture of females who perpetrate sexual offenses and their interactions with the criminal justice system. This knowledge can assist in prevention and intervention, thereby reducing female-perpetrated sexual offending and its impacts (Bickart et al., 2019).

The current study is distinctive in several ways. To date, a limited number of studies have explored the processing of female-perpetrated sexual offenses compared with male-perpetrated sexual offenses in the criminal justice system. Of the studies that have, most focus on sentencing with little focus on pretrial stages (e.g., arrest; Dara Shaw et al., 2022). Second, most studies rely mainly on bivariate analysis when exploring the differences in processing sexual offenses in the criminal justice system (Sandler & Freeman, 2011). In the present study, multinomial logistic regression was used, to determine whether the effects of perpetrator gender, age, and offense type predict the likelihood of law enforcement action. One of the greatest advantages of using multinomial logistic regression is that it offers predictive results (Nguyen & Park, 2021). In general, predictive modeling is argued to be highly beneficial when analyzing substantial amounts of data, as it allows for an objective appraisal of the data (Ting et al., 2018). Third, very few studies (and particularly those focused on female sexual offending) include juveniles (aged 10–17 years) who have perpetrated sexual offenses in their samples, despite prevalence studies on sexual victimization among adolescents indicating young people account for a sizable proportion of those inflicting the harm (Kjellgren, 2019).

Finally, and most importantly, most studies do not explore different offense types (Sandler & Freeman, 2011). In fact, to the authors’ knowledge, the current study is the first of its kind to explore the occurrence of reported sexual offending (against adults and children) *and* CSAM offenses across perpetrator gender and age, using police arrest records. Such information will specifically assist in addressing the significant gap in the literature on female-perpetrated offenses by providing a more detailed picture of who these females are, their prevalence and offending patterns, along with the influence of perpetrator gender and age, and the likelihood of action taken by law enforcement in response to these offenses. Also, the size of the current study’s sample, spanning nearly a decade, provides a unique opportunity to examine these patterns in more detail than ever before. Having a greater insight into this population should assist with prevention and intervention initiatives and, thereby, reduce female-perpetrated offending and associated impacts (Bickart et al., 2019).

## Methods

### Data

This research was approved by the researchers’ tertiary institution’s Human Research Ethics Committee. The current study utilized police arrest records obtained from one jurisdiction of Australia: the Queensland Police Service (QPS). The data was extracted from the Queensland Police Records and Information Management Exchange database. The dataset contained all sexual assault and related offenses actioned by the QPS between 1 January 2012 and 30 June 2021, resulting in 37,864 offenses. Offenses, where the perpetrator’s gender was not stated, were removed from the dataset (*n* = 23). The final sample comprised 37,841 offenses.

#### Perpetrator Age

Age was categorized into two groups: (a) juvenile (aged 10–17 years), and (b) adult (18+ years).

#### Offense Type

Offenses were classified into four categories based on victim age and offense setting, including (a) offline child sexual abuse (CSA) offenses, (b) offline sexual assault offenses, (c) online assaultive CSAM offenses (hereinafter referred to as assaultive CSAM offenses), and (d) online non-assaultive CSA offenses (hereinafter referred to as non-assaultive CSAM offenses). Offline CSA offenses included carnal knowledge of children, non-aggravated assault against a child, and non-assaultive sexual offenses and sexual assault offenses against a child victim. Offline sexual assault offenses included non-assaultive sexual offenses and sexual assault offenses against an adult or a victim of an unspecified age. Assaultive CSAM offenses included CSAM and involving a child in making CSAM. Non-assaultive CSAM offenses included using the internet to procure a child and procure/expose a child to an indecent act or matter offenses. It is important to note that this study does not focus on unique occurrences (i.e., a unique occurrence could involve two or more different perpetrators, multiple victims, and multiple offenses). Rather, the current study solely explored *offenses* to ascertain the prevalence of sexual offenses.

#### Action Taken

Action taken by law enforcement (i.e., QPS) against perpetrators for an offense includes various interventions or engagements. This was grouped into the following actions: (a) arrested; (b) referred to court (including notice to appear, summons issued, summons served, warrant issued, and offender ex officio indictment); (c) police diversion (including community conference, infringement notice issued, offender dealt with by another agency, restorative justice referral, and caution); and (d) no action taken (including perpetrator bar to prosecution, perpetrator currently in imprisonment, perpetrator not in public interest, and perpetrator psychiatric committal). The following actions were excluded from all analyses of action taken as there was not enough evidence available or were outside of police control: (a) not stated (*n* = 963), (b) perpetrator died (*n* = 308), (c) juvenile victim offense not disclosed at interview (*n* = 751), (d) juvenile victim offenses cannot be particularized (*n* = 739), and (e) juvenile victim too young without corroboration (*n* = 245). For all analyses, including action taken, the sample comprised 34,835 offenses.

### Analytical Approach

#### Preliminary Descriptive Statistics

Chi-square tests were conducted to explore the relationship between offense type and perpetrator characteristics (e.g., gender and age). A *p*-value of <.05 was used to indicate statistically significant associations between variables, and Cramer’s V (ϕ_*c*_) was used to interpret effect sizes. Post-hoc analyses, using adjusted standardized residuals greater than 2, were used to indicate significant differences between groups.

#### Main Analyses

Multinomial logistic regressions were used to determine if the interaction between perpetrator gender and age could predict the likelihood of offense type; and if the interaction between perpetrator gender, age, and offense type, could predict the likelihood of action taken (1 = *arrested*; 2 = *referred to court*; 3 = *police diversion*; 4 = *no action taken*). All assumptions were met, and a *p*-value of <.05 was used to indicate statistically significant findings.

## Results

### Prevalence of Sexual Offending

**Figure 1.**
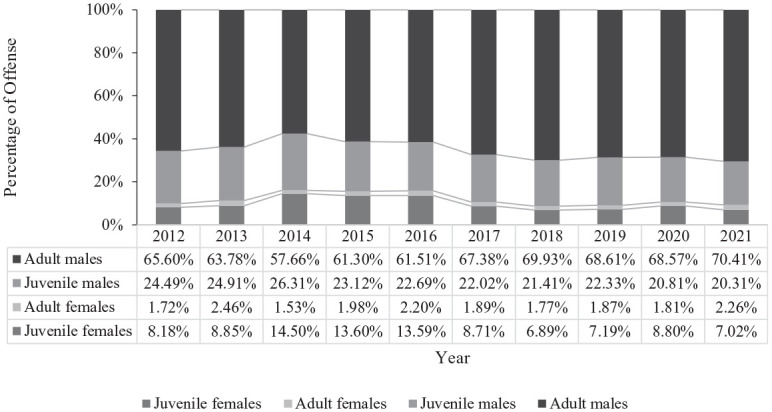
Prevalence of offenses by gender and age over time.

Most sexual offenses were perpetrated by males (87.8% of offenses; *n* = 33,233) as opposed to females (12.2% of offenses; *n* = 4,608). On the whole, the majority of the sexual offenses were perpetrated by adults (66.8%) compared with juveniles (33.2%), particularly by adult males (64.8% of all sexual offenses; 73.8% of all male-perpetrated sexual offenses). In instances where females had offended, juvenile females were responsible for the majority of these offenses (10.2% of all sexual offenses; 84.1% of all female-perpetrated sexual offenses). Figure 1 displays the percentage of offenses over time by gender and age.

There was a moderately, statistically significant association between gender and offense type, $\chi^{2}$ (3) = 5,851.98, *p* < .001, ϕ_*c*_ = .393. As shown in Table 1, almost three-quarters of offenses perpetrated by females were assaultive CSAM offenses. Males had more diversity in their offending; over three-quarters of their offenses were offline, split across offline CSA offenses and offline sexual offenses.

**Table 1.** Frequency (Percentage) and Adjusted Residuals of Offense Type Across Gender.

Offense Type	Females		Males	
	Frequency (Percentage)	Adjusted Standardized Residuals	Frequency (%)	Adjusted Standardized Residuals
Offline CSA offenses	966 (20.96%)	−38.4	16,997 (51.14)	38.4
Offline sexual offenses	224 (4.86%)	−31.7	8,611 (25.91)	31.7
Assaultive CSAM offenses	3,367 (73.07%)	76.2	6,695 (20.15)	−76.2
Non-assaultive CSAM offenses	51 (1.11%)	−6.8	930 (2.80)	6.8
Total	4,608 (100%)		33,233 (100)	

*Note*. CSA = child sexual abuse; CSAM = child sexual abuse material.

#### Juveniles

**Figure 2.**
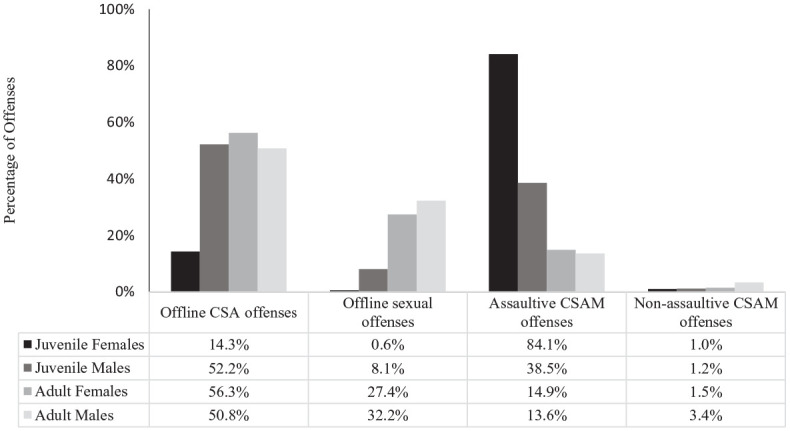
Percentage of offenses by perpetrator gender, age, and offense type.

As indicated in Table 2 and Figure 2, there was a moderately, statistically significant association between gender and offense type for juveniles, $\chi^{2}$ (3) = 2,271.16, *p* < .001, ϕ_*c*_ = .425. Juvenile females predominantly came to police attention for assaultive CSAM offenses, followed by offline CSA offenses. Juvenile males appeared to have more diversity in their offending, and mostly perpetrated offline CSA offenses followed by assaultive CSAM offenses.

**Table 2.** Frequency (Percentage) and Adjusted Residuals of Offense Type Across Juvenile Males and Females.

Offense Type	Juvenile Females		Juvenile Males	
	Frequency (Percentage)	Adjusted Standardized Residuals	Frequency (%)	Adjusted Standardized Residuals
Offline CSA offenses	553 (14.27%)	−40.0	4,543 (52.20)	40.0
Offline sexual offenses	23 (0.59%)	−16.6	702 (8.07)	16.6
Assaultive CSAM offenses	3,258 (84.10%)	47.3	3,353 (38.53)	−47.3
Non-assaultive CSAM offenses	40 (1.03%)	−0.8	105 (1.21)	0.8
Total	3,874 (100%)		8,703 (100)	

*Note*. CSA = child sexual abuse; CSAM = child sexual abuse material.

#### Adults

As indicated in Table 3 and Figure 2, there was a minimal, statistically significant association between gender and offense type for adult perpetrators, $\chi^{2}$ (3) = 17.74, *p* < .001, ϕ_*c*_ = .026. Although a statistically significant difference was found, the effect size was small, with both adult males and females mostly arrested for offline offenses (83.01% and 83.65%, respectively). Adult females mostly perpetrated offline CSA offenses followed by offline sexual offenses. The majority of adult males also mainly perpetrated offline CSA offenses, but had a lower percentage, followed by offline sexual offenses.

**Table 3.** Frequency (Percentage) and Adjusted Residuals of Offense Type Across Adult Males and Females.

Offense Type	Adult Females		Adult Males	
	Frequency (Percentage)	Adjusted Standardized Residuals	Frequency (%)	Adjusted Standardized Residuals
Offline CSA offenses	413 (56.27%)	2.9	12,454 (50.77)	−2.9
Offline sexual offenses	201 (27.38%)	−2.8	7,909 (32.24)	2.8
Assaultive CSAM offenses	109 (14.85%)	1.0	3,342 (13.62)	−1.0
Non-assaultive CSAM offenses	11 (1.50%)	−2.8	825 (3.36)	2.8
Total	734 (100%)		24,530 (100)	

*Note*. CSA = child sexual abuse; CSAM = child sexual abuse material.

### Multinomial Logistic Regression Results

#### Does Perpetrator Gender and Age Predict Offense Type?

A multinomial logistic regression was performed to determine whether the interaction between perpetrator gender and age could predict the type of offense perpetrated (see Table 4). The logistic regression model was statistically significant, $\chi^{2}$ (9) = 10,433.87, *p* < .001.

**Table 4.** Multinomial Logistic Regression Predicting the Likelihood of Offense Perpetrated based on the Interaction Between Gender and Age: Relative Risk Ratios (95% CI).

Interaction Between Gender and Age	Offline Sexual Offenses	Assaultive CSAM Offenses	Non-Assaultive CSAM Offenses
Adult males	15.27 (10.05, 23.19)*	0.05 (0.04, 0.05)*	0.92 (0.66, 1.27)
Adult females	11.70 (7.46, 18.35)*	0.05 (0.04, 0.06)*	0.42 (0.19, 0.73)*
Juvenile males	3.72 (2.43, 5.68)*	0.13 (0.11, 0.14)*	0.32 (0.22, 0.47)*

*Note*: The reference category is offline CSA offenses and juvenile females. CI = confidence interval; CSA = child sexual abuse; CSAM = child sexual abuse material.

* *p* < .01. *N* = 37,840.

The odds of perpetrating an offline sexual offense were 15.27 times greater for adult males, 11.70 times greater for adult females, and 3.72 times greater for juvenile males, compared to juvenile females. The odds of perpetrating an assaultive CSAM offense were 20 times higher for juvenile females compared to both adult males and adult females, and 7.69 times greater for juvenile females compared to juvenile males. The odds of perpetrating a non-assaultive CSAM offense were 2.38 and 3.13 times greater for juvenile females compared to adult females and juvenile males, respectively. Interestingly, there was no significant difference in the odds of perpetrating a non-assaultive CSAM offense for male adults and juvenile females.

#### Does Perpetrator Gender, Age, and Offense Type Predict the Likelihood of Action by Law Enforcement?

A multinomial logistic regression was performed to determine whether the effects of offense type and the interaction between perpetrator gender and age could predict action taken (arrested, referred to court, police diversion, and no action taken, see Table 5). The logistic regression model was statistically significant, $\chi^{2}$ (18) = 27,215.68, *p* < .001.

**Table 5.** Multinomial Logistic Regression Predicting the Likelihood of Action Taken Based on Offense Type and the Interaction Between Gender and Age: Relative Risk Ratios (95% CI).

Variables in the Model:	Arrested	Referred to Court	Police Diversion
Gender and age: reference category is adult males			
Juvenile females	0.02 (0.01, 0.02)*	0.02 (0.01, 0.03)*	11.23 (9.36, 13.48)*
Juvenile males	0.11 (0.10, 0.12)*	0.10 (0.08, 0.17)*	14.22 (12.41, 16.29)*
Adult females	0.47 (0.35, 0.62)*	0.54 (0.39, 0.74)*	1.54 (1.02, 2.30)*
Offense type: reference category is offline CSA offenses			
Non-assaultive CSAM	2.66 (1.80, 3.94)*	2.86 (1.88, 4.35)*	3.54 (26, 5.54)*
Assaultive CSAM	1.50 (1.30, 1.72)*	2.27 (1.94, 2.65)*	7.17 (6.26, 8.21)*
Offline sexual offenses	2.04 (1.78, 2.34)*	3.18 (2,74, 3.69)*	3.13 (2.63, 3.72)*

*Note.* CI = confidence interval; CSA = child sexual abuse; CSAM = child sexual abuse material.

* *p* < .05. *N* = 34,834; reference category: no action taken.

The odds of being arrested were 98% lower for juvenile females, 89% lower for juvenile males, and 53% lower for adult females, compared to adult males. The odds of being arrested were 2.7 times greater for non-assaultive CSAM offenses, 1.5 times greater for assaultive CSAM offenses, and 2.04 times greater for offline sexual offenses, compared to offline CSA offenses.

The odds of being referred to court were 98% lower for juvenile females, 90% lower for juvenile males, and 46% lower for adult females, compared to adult males. The odds of being referred to court were 2.9 times greater for non-assaultive CSAM offenses, 2.3 times greater for assaultive CSAM offenses, and 3.2 times greater for offline sexual offenses, compared to offline CSA offenses.

The odds of receiving police diversion were 11.2 times more likely for juvenile females, 14.2 times more likely for juvenile males, and 1.5 times more likely for adult females, compared to adult males. The odds of receiving police diversion were 3.5 times more likely for non-assaultive CSAM offenses, 7.2 times more likely for assaultive CSAM offenses, and 3.1 times more likely for offline sexual offenses, compared to offline CSA offenses.

## Discussion

This study aimed to further current empirical knowledge of female-perpetrated sexual offenses, including CSAM offenses, that enter the criminal justice system. In particular, the focus was on learning more about the prevalence of sexual offenses and types of offenses perpetrated; the relationship between perpetrator gender, age, and offense type; and the relationship between perpetrator gender, age, offense type, and likelihood of action taken by law enforcement. The size of the sample, spanning nearly a decade, provided a unique opportunity to examine offense patterns in more detail than has been done before. To the authors’ knowledge, this was the first study to explore the occurrence of reported sexual offenses (against adults and children) *and* CSAM offenses, comparing perpetrator gender and age, using policing data. Key findings from the current study provide critical information that can be used to inform understanding of sexual offending behavior perpetrated by both males and females, and adults and young people, and help to inform prevention and intervention strategies. These findings, limitations of the current study, implications, and areas for future research are discussed below.

Consistent with other official statistics (Australian Bureau of Statistics [ABS], 2022) adults were responsible for the majority of sexual offenses in this sample, with adult males being the main perpetrators of these offenses. However, compared with earlier research, the proportion of sexual offenses perpetrated by females within this examined period appears to have increased. While no two samples are the same across studies, this rate is much higher than in previous research that has utilized police data. For example, Cortoni et al. (2016) found only 2.2% of sexual offenses reported to the police were committed by females. In a different study that explored sexual incidents reported to the police, only 5.4% involved females (Williams & Bierie, 2015). The rate of female-perpetrated sexual offenses identified in the current study is more similar to the rate identified in victimization studies (Mdn = 10.8%; Cortoni et al., 2016).

One reason for this finding might be due to the data collection periods. Cortoni et al.’s (2016) research spanned from 2000 to 2013 and Williams and Bierie’s (2015) study covered 1991 to 2011, whereas the current study used data from 2012 to 2021. Since these earlier periods, there have been positive advancements in the way female-perpetrated sexual offending is reported in the media, stepping away from undertones of sympathy and romanticization (Christensen, 2018b). This change in reporting could potentially have contributed to altering public perceptions (Christensen, 2018b), and as a result, more individuals reporting female-perpetrated sexual violence to authorities. In fact, Australian data reveals an almost 208% increase in the number of females being proceeded against for sexual assault as their main offense from 2008 (*n* = 222) to 2023 (*n* = 683; ABS, 2024).

The current findings indicate that the higher rate of female-perpetrated sexual offenses appears to be mostly attributable to the proportion of sexual offenses committed by juvenile females that came to the attention of the police (84.1% of all female-perpetrated sexual offenses), and notably for assaultive CSAM offenses. Additional analyses reaffirmed delineations in offending by perpetrator gender and age, most evident among the juvenile sample. Although there was a minimal, statistically significant association between gender and offense type for adult perpetrators, moderate, statistically significant differences were found for juveniles. Juvenile females mostly perpetrated assaultive CSAM offenses, whereas, juvenile males appeared to have more diversity, and mostly perpetrated offline CSA offenses followed by assaultive CSAM offenses. The findings regarding female-perpetrated offenses are likely related to digital media advancement and young people’s rapid transition to online social (and sexual) interactions over the past decade, along with the issue of juvenile females being over twice as likely as juvenile males to have been invited to send self-generated material (22% vs. 8%; U.K. Safer Internet Centre et al., 2017). This transition has created an emerging, and concerning, phenomenon of young females engaging in online harmful sexual behavior, leading to law enforcement action.

Two earlier studies spanning 2000 to 2013 (Cortoni et al., 2016) and 1991 to 2011 (Williams & Bierie, 2015) that also included young people, reinforce these potential changes in social behavior among juveniles. For example, Cortoni et al. (2016) found that female-perpetrated sexual violence among juvenile females was only slightly higher than among adult females (2.3% vs. <2%, respectively). In contrast, the current study found sexual violence amongst juvenile females to be a lot higher than among adult females (10.2% vs. 2%, respectively). The current finding is in line with Napier (2022) who recently found that the age of one being first exposed to CSAM has decreased over time, with more young people exploring this material compared with prior generations. Napier (2022) argues that engagement with self-generated material (i.e., sexting) might normalize the viewing of CSAM, and conversely, the viewing of CSAM might normalize self-generated material. Aside from research indicating that juvenile females are vulnerable to engaging with self-generated material compared with juvenile males (e.g., Napier, 2022; U.K. Safer Internet Centre et al., 2017), to the authors’ knowledge, there is no comparable literature to help elucidate the patterns of offense perpetration across female and male juveniles found in the present study. Nonetheless, the findings highlight an emerging phenomenon of young females engaging in online harmful sexual behaviors, resulting in criminal justice intervention. These findings could also be related to CSAM offenses occurring within intimate relationships (domestic and family violence) as a result of coercive methods. It is also acknowledged that a history of trauma (including domestic violence and child abuse) may be associated with offending (Levenson et al., 2015).

It was interesting to find that the odds of perpetrating an online assaultive CSAM offense were 20.0 times higher for juvenile females compared to both adult males and adult females, and 7.7 times higher for juvenile females compared to juvenile males. Additionally, the odds of perpetrating a non-assaultive CSAM offense were 2.4 and 3.1 times greater for juvenile females compared to adult females and juvenile males, respectively, but not significantly different from adult males. Again, while further research is much needed, it could be that a lot of the assaultive CSAM concerning juvenile females is self-generated and could be the product of coercive victimization, particularly as juvenile females are at a 70% higher risk of coercive victimization compared with males (Kernsmith et al., 2018). However, it should not be disregarded that a proportion of these assaultive CSAM cases could have involved other children being victimized in the CSAM (as opposed to solely being self-generated material), potentially highlighting some ill-intent motivation.

Several implications arise from these key findings. From a public health approach to sexual violence and abuse prevention (e.g., McKillop, 2019; Quadara, et al, 2015; Smallbone & Rayment-McHugh, 2017), it is evident that tailored primary- and secondary-level prevention strategies are required to address the problem of sexual violence and abuse, and reduce the extent of these offenses. We do, however, acknowledge that the police service in this jurisdiction aims to adopt an educative and prevention approach in situations involving similarly aged young people who engage in sexting (QPS Operational Procedures Manual s 7.11.3, 2023). As discussed, juvenile males had more diversity in their perpetration, whereas juvenile females appeared to be more concentrated in their perpetration with assaultive CSAM offenses. These different patterns in perpetration suggest different responses to prevent and intervene with youth might be required, allowing for more tailored interventions to effectively address this social problem. For example, gender-specific education programs for juvenile females might predominantly focus on preventing online harmful sexual behaviors, emphasizing the risks of self-generated images as well as sharing sexual images online with others, whereas gender-specific education for juvenile males could focus mostly on preventing in-person harmful sexual behaviors and the contextual features that increase likelihood of offending. Certainly, gender-specific education programs could begin in late elementary school, to help raise awareness of the risks—particularly on online platforms—from an early age, alongside other community awareness-raising strategies.

Law enforcement could be involved in the augmentation of such primary and secondary prevention efforts (e.g., awareness-raising and education campaigns) in collaboration with schools and community groups focused on this prevention; as detecting and disrupting CSAM is not sufficient to prevent children from associating with CSAM (Napier, 2022), nor may it be the most appropriate measure to respond to many of these offenses. In turn, law enforcement agencies might need to reconsider current responses, and the role of education and diversion strategies, where appropriate, to reduce contact with the criminal justice system and encourage desistance. However, the first crucial step is to learn more about this group through further research.

Future research needs to explore why these juvenile females are engaging in such a concentration of CSAM perpetration. For example, whether most of this material is self-generated and, if so, whether the material is produced willingly, or if the young person is coerced. This research would require a gendered lens given the current findings and also that research has found girls are most vulnerable to engaging with self-generated material (U.K. Safer Internet Centre et al., 2017). Future research also needs to explore the relationships between the perpetrator and victim to learn more about this group; for example, whether these assaultive CSAM cases perpetrated by juvenile females occur mostly between peers (e.g., sexting) or whether they involve unknown victims, and the specific types of CSAM that they are possessing, distributing, and producing. Furthermore, research is required to explore whether these offenses are occurring by one perpetrator or co-perpetrators to understand the additional dynamics involved; as well as the motivations behind juvenile females engaging in this form of offending to inform primary and secondary prevention efforts in line with a public health approach to CSA prevention.

In terms of criminal justice system responses (i.e., tertiary prevention), a final key finding was in relation to perpetrator gender, age, and offense type predicting the likelihood of law enforcement action taken; for all sexual offense types the requirement for further law enforcement action and the gravity of these actions (e.g., arrest and referral to court), were significantly lower among all female perpetrators (both juvenile and adult) and juvenile males compared with adult male perpetrators. In particular, the multinomial logistic regression revealed that these juvenile females were least likely of all groups to have any serious action taken (i.e., arrested or referred to court). This finding was not surprising and is consistent with the literature, particularly with the disparity evident in the sentencing of offenders, with females receiving less punitive outcomes than males (e.g., Beeby et al., 2020; Sandler & Freeman, 2011). While certain theories outlined in previous literature might account for the discrepancies, such as the chivalry theory (as cited in Nagel & Hagan, 1983) or cognitive dissonance (see Damiris et al., 2021), it could also be that the actions taken by law enforcement relate to the *context* of the offending behavior; that is, the engagement with self-generated material (i.e., sexting) and police attempting to navigate how to most suitably process these young females being caught up in harmful online sexual behavior.

Regardless of the reason, there is a need to ensure these potentially more lenient outcomes are not harmful to any victims involved, with their experiences being minimized or invalidated (Damiris et al., 2021); and ensure accountability for these actions. Further, consideration should be given to whether there are currently missed opportunities to deter offending. For example, a diversion program for young people specifically focused on CSAM could be introduced that offers education and information to address perpetration (and potential future perpetration), to reduce entrenchment in the system. While there is some evidence demonstrating the effectiveness of diversion programs for juveniles (e.g., justice-involved youth with behavioral health issues; Kretschmar et al., 2018), many considerations need to first take place prior to implementation, along with further research and pilot programs. For those who are subsequently found guilty of sexual offenses, there are some treatment services offered within the jurisdiction to address re-offending. To assist with ensuring the most effective law enforcement actions are taken, future research needs to explore why we are seeing this disparity in action taken, particularly across male and female juveniles.

### Limitations

The current study is not without limitations. First, the current study uses police data and therefore is not representative of the true extent of female-perpetrated sexual violence in our society. However, the sample is a very large size and spans almost a decade, offering an impressive sample to explore these patterns. Second, although this dataset represents all cases reported to the police and does not exclude, for example, any ethnicity, socioeconomic status, or religion, our focus on gender, and age constrained a more nuanced examination of these additional factors which remain crucial for informing research and practice moving forward. Future diversity-related research, particularly on the intersectionality of certain characteristics such as cultural background and gender and how this might influence offending and law enforcement responses, would be beneficial. Other diversity-related research on this topic should include a focus on the LGBTIQ+ community, which often have elevated rates of sexual victimization (Katz-Wise & Hyde, 2012; Rothman et al., 2011), along with a focus on those in remote regions where access to technology might differ. Doing so will provide an even more nuanced examination that brings a stronger intersectionality lens to this topic, particularly for vulnerable groups, assisting with more tailored prevention and intervention efforts. Replication of findings in other Australian and international jurisdictions is also warranted to validate these patterns and enable generalizability.

Third, given database constraints, we were unable to capture additional various characteristics (e.g., victim-perpetrator relationship and criminal history). Such information would have provided a much fuller picture of offending dynamics and allow for more sophisticated analyses to control for variables such as offending history when examining law enforcement outcomes. Fourth, the analyses were incident-based. As such, chronicity in offending by unique individuals was not measured making it difficult to discern the overall proportion of offending per unique individual—another important avenue for future research. As aforementioned, this study looked at all offenses rather than unique occurrences, which could potentially artificially inflate results. Future research could instead focus on offenders, rather than offenses. Further, law enforcement actions against offenders for offenses were categorized into arrest, initiation of prosecutions, police diversion, and taking no action, therefore the data does not recognize civil action taken, namely police protection notice/domestic violence order (where a relevant relationship—intimate personal, family, and informal care—exists between the perpetrator and the victim). This could be one avenue for future research. Whilst the relationship of co-perpetrators was out of scope for the current paper, it is a vital area for future research that may provide a deeper understanding of female-perpetrated CSA and CSAM offenses. Nonetheless, the current study has provided a solid starting point and has highlighted several new emerging areas for future research.

## Conclusion

Improving knowledge of female-perpetrated sexual violence is vital to inform prevention and intervention efforts. The findings bring attention to the different patterns of sexual violence stratified by gender and age, which means tailored responses are likely required across these groups to address the problem of sexual violence and abuse. Despite the identified limitations of the current study, several implications arose, specifically, primary- and secondary-level prevention strategies. With the apparent growth in the number of sexual offenses committed by juvenile females engaging in online harmful sexual behavior, gender-specific education programs that begin from an early age (e.g., late elementary school) and raise awareness of the risks online alongside other community awareness-raising strategies, might be beneficial. Programs for juvenile females could potentially include the risks of self-generated images and sharing such images, while programs for juvenile males could focus on the prevention of in-person harmful sexual behaviors. However, before introducing this primary-level prevention strategy, further investigation to inform proactive strategies and effective law enforcement responses is warranted. In terms of secondary-level prevention, a diversion program for young people specifically focused on CSAM could, potentially, address some perpetration (and potential future perpetration) through education and information. Given the lack of research on female-perpetrated sexual violence, the global proliferation of CSAM, and the dearth of literature on female-perpetrated CSAM offenses, the current findings provide a seminal platform for further research, policy, and practice development in this space.
